# A study of parental decision-making over the vaccination of girls, based on the protection motivation theory and the elaboration likelihood model

**DOI:** 10.3389/fpubh.2022.1024399

**Published:** 2022-11-10

**Authors:** Qi Wang, Fangzhou Zhou, Wen Zhang, Chenjin Tang

**Affiliations:** ^1^School of Industrial Design, Hubei University of Technology, Wuhan, China; ^2^Institute of Communication Studies, Communication University of China, Beijing, China; ^3^School of Journalism and Culture Communication, Zhongnan University of Economics and Law, Wuhan, China

**Keywords:** protection motivation theory (PMT), elaboration likelihood model (ELM), parental decision-making, HPV vaccine, secondary risks

## Abstract

This study proposed a new theoretical framework that combines the protection motivation theory and the elaboration likelihood model to examine how health information processing patterns influence parents' vaccination decision-making on behalf of their daughters. Based on survey data from 359 parents of girls aged 9–15, we tested the theoretical model by using structural equation model. The results showed that the central route, represented by information quality, affected the parents' perceptions of HPV severity and susceptibility; the peripheral route, represented by source credibility, influenced their perceptions of HPV severity, HPV susceptibility, vaccine response efficacy, and secondary risks. Also, Chinese parents' perceptions of HPV vaccines, not perceptions of HPV, affected their intention to vaccinate their daughters. The study suggests in addition to improving the quality of health information, the peripheral route, such as the release of vaccination photos, public immunization evaluations, and case narratives, should also be used to change parents' perceptions. Besides, reducing the traditional stigmatization of female sexuality and improving parents' understanding of the new generation's sexual attitudes will increase parents' intention to have their daughters vaccinated against HPV.

## Introduction

Although HPV vaccination can considerably decrease the incidence of high-risk HPV infections (1), the rate of early vaccination with HPV vaccines in China is rather low (2). Less than 1 percent of school-age girls (9–15 years) receive HPV vaccination (3). According to the *China Family Development Report*, adolescents' first sexual experience takes place on average at the age of 15.9 years, and in recent years this average has trended upward (4). Early HPV vaccination can help prevent reproductive disorders and preserve women's health.

Parents play an essential role in whether girls aged 9–15 are vaccinated. HPV vaccine-related health information changes parents' perceptions of the vaccines and is critical to parents' decisions on behalf of their children (5). It can reduce parents' negative attitudes toward HPV vaccines (6) and facilitate their decisions to vaccinate their daughters against HPV (7).

Nowadays, social media have become a significant channel for delivering HPV vaccine information, altering people's information processing patterns (8). Unintended media exposure has increased, which can produce different information processing patterns than deliberate media exposure (9). For example, when people search for information about a specific health problem, they are more inclined to consider its meaning deeply, engaging the “central” route of elaboration. In contrast, when people stumble upon health information on TikTok, they may only elaborate superficially on a message *via* the “peripheral” route. In addition, the different forms of information online lead to different information processing routes (10). For instance, if health information is hard to comprehend or of low quality, individuals may activate the “peripheral” rather than the “central” route to process it. However, how individuals' health information processing alters the effect of this information remains a “black box.”

This study assesses how the two routes of information processing influence perceptions of vaccine efficacy and other factors motivating child-protective behavior. We apply the protection motivation theory (PMT) to vaccination behavior and position it within the framework of the elaboration likelihood model (ELM) to derive hypotheses about the effects of different information processing routes using data from Chinese parents of girls aged 9–15.

Theoretically, the study explains the influence of parents' health information processing patterns on the health decisions they make for their children; it finds a shift in the explanatory model of media effects from media exposure to media information processing. Empirically, the quality and sources of information are also likely to influence child-protective behavior; thus, health organizations should focus on providing high-quality information and countering online disinformation through credible media.

### The protection motivation theory and the motivation for parental protective behaviors

PMT has been frequently used in recent years to explain people's attitudes and behaviors toward vaccination (11). The theory has been abstracted into three parts: information sources, cognitive mediating processes, and coping modes (12). Individuals form cognitions through information sources, determine whether to form protective motives based on cognitive mediating processes, and subsequently decide which coping mode to adopt (13).

PMT has been widely used in health behavior studies. Studies have examined the relationship between various elements of PMT and treatment behaviors in the areas of pregnancy exercise (14), malaria (15), cancer (16), adolescent smoking behavior (17), vaccination (18), and physical activity (19).

PMT emphasizes the role of cognitive mediating processes in health behavior change. Threat appraisal and coping appraisal are initiated when people confront information sources (20). Threat appraisal refers to the individual's assessment of a threat's severity and susceptibility. Coping appraisal refers to an individual's assessment of response efficacy, self-efficacy, and response cost (the cost of practicing the health behavior) (12).

HPV vaccination behaviors are influenced by perceived HPV severity, perceived HPV susceptibility, and vaccine response efficacy from PMT (21). These also affect the motivation for HPV vaccination among men who have sex with men (22). Self-efficacy, as an important component in PMT, is mainly involved in long-term health behaviors such as exercise (23), skin protection (24), and quitting smoking (25), yet HPV vaccination is an immediate health behavior based on vaccination decision-making. Therefore, we did not contain self-efficacy as a variable in the study. Generally speaking, the motivation for vaccination will be strongest when individuals consider the health threat to be severe and perceptible and the promoted health behavior to be effective at reducing the threat.

However, PMT does not consider the current risk culture, where people adopt protective behaviors while worrying about a range of secondary risks associated with the protective behaviors. Pawlowski (26) argued that while child seats can protect infants in car crashes, parents are also concerned that these seats increase the risk of sudden infant death while driving. The direct risk that arises from implementing risk-responsive behaviors is referred to as secondary risk, a term that was first used in the field of project risk management (27). Cummings et al. (28) validated and compared the effectiveness of secondary risk theory and PMT in explaining the inoculation rates of four vaccines. They found that when news messages emphasized vaccines' side effects, the secondary risk theory had more explanatory power than PMT.

In China, the news is often criticized for acting as a “magnifying glass” for vaccine risks (29). The news emphasizes episodic vaccine events, magnifying the public's perception of vaccine risks. After a hepatitis B vaccine incident in 2013, for example, reports from 10 provinces and cities showed that the national hepatitis B vaccination rate dropped by 30% within 1 month (30). We infer that when parents are deciding whether to have their daughters vaccinated against HPV, they assess the threat, response efficacy, and secondary risks simultaneously. The perceptions of HPV threat and the efficacy and risks of HPV vaccines affect parents' willingness to vaccinate their children, respectively. The absence of any of these factors may reduce protection motivation; thus, we make the following hypotheses.

*H1a: Perceived HPV severity is positively associated with parents' intention to vaccinate their daughters against HPV*.
*H1b: Perceived HPV susceptibility is positively associated with parents' intention to vaccinate their daughters against HPV*.
*H1c: Perceived response efficacy of HPV vaccines is positively associated with parents' intention to vaccinate their daughters against HPV*.
*H1d: Perceived secondary risk is negatively associated with parents' intention to vaccinate their daughters against HPV*.

### Influence of health information processing on parents' perceptions of protective behaviors

The media ecosystem is considered an information source in the PMT, influencing individuals' cognitive mediation process. Individuals generally do not have a systematic knowledge of viruses or vaccines, and the media has become the main channel for individuals to acquire health knowledge (31, 32) and profoundly affect people's knowledge and perceptions of viruses and vaccines (33). The media thus plays a key role in risk explanation (34). The risks that people perceive are “pseudo-risks” that have been selected, processed, and reconstructed through the media (35). Cognitive processes are the theoretical core of PMT, and information sources are often under-researched in model validation (36).

Although media exposure profoundly influences people's cognitive mediation processes and subsequent health-protective behaviors as an information source, how individuals process the health information they encounter and the effects of information processing patterns remain unknown (37). Petty and Cacioppo (38) proposed the ELM, which explains audience attitude changes based on two routes of information processing: central and peripheral. The central route induces a high level of message processing, and the information effect is the result of thoughtful consideration of arguments related to the quality of the message. The peripheral route induces a low level of message processing, and attitudes are changed through heuristic cues and contextual factors related to the content of the message (39).

When individuals consider health information in depth, the information is often processed through the central route in a way that produces a stronger persuasive effect. In this case, individuals' assessment of health information quality occurs through the central processing route, which significantly influences their willingness to adopt the information (40). Accuracy, relevance, and completeness are used to measure the perceived quality of social media health knowledge, and information quality is the key to stimulating the central route (41). In previous studies, information quality heightens older people's threat perceptions (i.e., severity and susceptibility) of coronavirus disease 2019 (42). Inaccurate information from blog comments aggravates parents' misunderstanding of the risks of vaccines (43).

However, individuals do not always process health information thoughtfully. They may be exposed to it passively or unconsciously (44), such as by occasionally glancing at health information while browsing TikTok, Twitter, or Facebook for entertainment. When other matters occupy individuals' cognitive resources, any changes in attitudes or perceptions are mainly accomplished through the peripheral route, and perceived source credibility is a common research variable. For example, Shang et al. (45) took source credibility as an indicator of the peripheral route to observe the social media health information sharing behaviors among seniors. Chen and Pan (46) measured source credibility through the reputation of short video platforms and the credibility of video creators in their evaluations of the effect of short videos. Source credibility can influence the bias effect of narrative information on the perceptions of vaccination risks (47). It can also affect the compliance of health information through the mediation of perceived threat and perceived efficacy (48).

When parents process HPV-related health information through the central route, they focus on information quality. However, when parents do not expend efforts to obtain high-quality information or fail to assess information quality, source credibility becomes the most commonly used peripheral cue to influence their perceptions. The underestimation of HPV threats and the perceived vaccine risks mainly come from misleading information or the inherent impression from previous vaccine risk events (49). When parents accept relevant health knowledge or trust the information source, they can recognize the HPV threats and vaccine efficacy and lower the risk perceptions of HPV vaccines. Therefore:

*H2a: Information quality is positively associated with parents' perception of HPV severity*.
*H2b: Information quality is positively associated with parents' perception of HPV susceptibility*.
*H2c: Information quality is positively associated with parents' perception of the response efficacy of HPV vaccines*.
*H2d: Information quality is negatively associated with parents' perception of the secondary risk of HPV vaccination*.
*H3a: Source credibility is positively associated with parents' perception of HPV severity*.
*H3b: Source credibility is positively associated with parents' perception of HPV susceptibility*.
*H3c: Source credibility is positively associated with parents' perception of the response efficacy of HPV vaccines*.
*H3d: Source credibility was negatively associated with parents' perception of the secondary risk of HPV vaccination*.

### Theoretical framework

**Figure 1:**
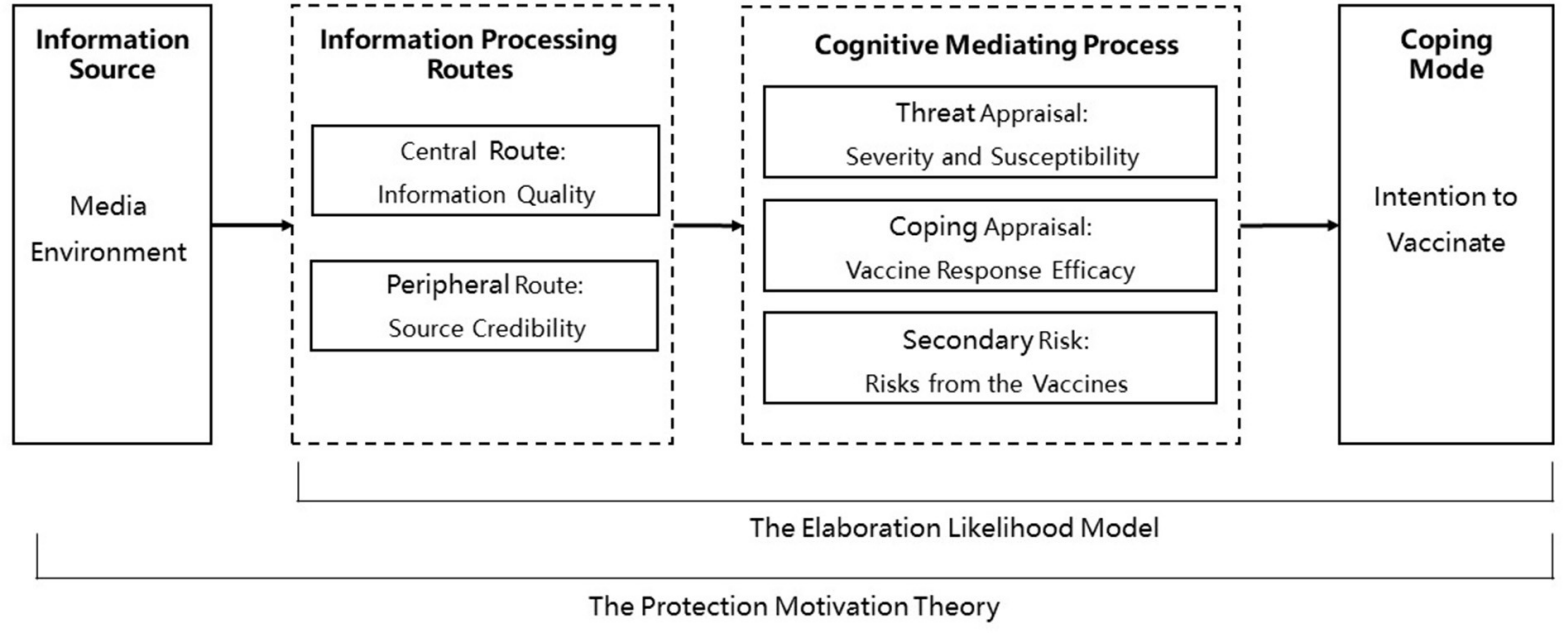
Research framework.

This study combines the ELM and the PMT to explain how parents' processing of HPV-related health information affects their protection motivation and thus influences their decision to immunize their daughters against HPV. In the integrated model, parents in the pseudo-environment constructed by the media (information source) activate either the central route (information quality assessment) or the peripheral route (source credibility assessment) for processing HPV-related information. Both processing routes may influence their threat perceptions of HPV, response efficacy perceptions of the vaccine, and risk perceptions (cognitive mediating processes), ultimately influencing their decisions whether to have their daughters vaccinated against HPV (coping mode). The study framework is shown in Figure 1: information quality and source credibility are independent variables; perceived HPV severity, susceptibility, vaccine response efficacy, and secondary risk are mediating variables; and parents' intention to vaccinate their daughters is the dependent variable.

## Methods and measures

We selected three primary and four junior high schools in Hubei Province by stratified sampling and then 24 classes from Grades Four through Nine (four classes per grade) by generating random numbers. Questionnaires were distributed to the girls' parents, who filled them out on a volunteer basis; they were given a gift worth about 10 RMB as a small honorarium. We obtained informed consent forms from all the participants. This study was approved by the Research Ethics Committee of the corresponding's author's university.

The questionnaire was divided into two parts with a total of 43 items. The first part measured demographic variables, and the second part measured the research variables across seven dimensions: information quality as the central processing route, source credibility as the peripheral processing route, HPV severity, HPV susceptibility, secondary risk, vaccine response efficacy, and intention to vaccinate their daughters. The questionnaire was adapted from previous studies, and items were assessed on a 5-point Likert scale, with 1 = “strongly disagree” and 5 = “strongly agree.”

The information quality and source credibility scales were mainly drawn from Lim and Kim (50)'s questionnaire. Information quality was measured with 12 items (Cronbach α = 0.971) to assess parents' perceptions of the quality of HPV-related health information, and was subdivided into four dimensions: relevance (3 items, e.g., “*The online HPV and vaccine information is exactly what I need.”*), understandability (3 items, e.g., “*The online HPV and vaccine information is easy to read.”*), adequacy (3 items, e.g., “*The online HPV and vaccine information is complete.”*), and usefulness (3 items, e.g., “*The online HPV and vaccine information is helpful.”*). In the exploratory factor analysis, only one factor was extracted from the items of information quality. Therefore, these four dimensions did not appear as variables, respectively.

Source credibility (Cronbach α = 0.830) assessed parents' trust in the source of health information. The items included “*The information about HPV vaccination released by relevant government agencies (such as health commissions and local health bureaus) is very credible”* and “*The information about HPV vaccination released by the official media (such as CCTV News, Xinhua News, and People's Daily) is very credible.”*

The HPV severity and HPV susceptibility scales were based on Patty et al. (51)'s study. HPV severity was measured with five items (Cronbach α = 0.922) that evaluated parents' perception of the danger of HPV, such as “*HPV is scary to girls”* and “*HPV infection can have a serious impact on a girl's health.”*

HPV susceptibility was measured with two items (Cronbach α = 0.715) that assessed parents' perception of the probability of their daughters being infected with HPV. The items included “*HPV is highly contagious for girls”* and “*There is a high possibility of HPV infection after having sex for girls.”*

Secondary risk was measured with eight items (Cronbach α = 0.933), which focused on parents' perceptions of side effects of HPV vaccines and included two dimensions. Secondary severity was measured with four items such as “*Side effects of HPV vaccines can cause serious illness to girls”* and “*Side effects of HPV vaccines can affect girls' daily activities.”* Secondary susceptibility was measured with four items such as “*Girls who receive HPV vaccination are prone to side effects”* and “*Juveniles who receive HPV vaccination are prone to side effects.”*

Vaccine response efficacy was measured with three items (Cronbach α = 0.886) that examined parents' confidence in HPV vaccines' benefits, including “*HPV vaccines can increase girls' resistance to HPV”* and “*HPV vaccines can effectively prevent girls' genital warts, cervical cancer, and other diseases in future.”*

Intention to vaccinate was measured with three items (Cronbach α = 0.956) that assessed parents' consent regarding the HPV vaccination of their daughters. The scale was adopted from Yang (52), and included items such as “*I am considering getting my daughter vaccinated against HPV”* and “*I will get my daughter vaccinated against HPV according to the provided vaccination schedule.”*

In addition, demographic variables were included in the study as control variables, including parents' gender, age, place of residence, education, occupation, income, and number of children, and the ages of their daughters.

## Results

### Descriptive analysis

Response rate was 38.6 % and a total of 435 questionnaires were collected. After data screening, 76 invalid questionnaires were excluded because the answers to different questions were too similar, and 359 questionnaires were finally included in the data analysis. The sample consisted of 72 fathers (20.1%) and 282 mothers (78.6%), and 5 respondents did not provide their gender (1.4%). The mean age of the parents was 40.95 years old (SD = 4.05); 58.8% of the parents had only one daughter, 15.9% had two daughters, and 22.6% had one son and one daughter. The mean age of the daughters was 12.96 years old (SD = 1.40). 93.3% of the parents were currently living in an urban area.

The results of the descriptive analysis are shown in Table 1. The parents perceived the quality of online information about HPV and vaccines as moderate (M = 3.31, SD = 0.72 for fathers; M = 3.42, SD = 0.67 for mothers), yet they showed a high degree of trust in the source credibility (M = 4.62, SD = 0.46 for fathers; M = 4.49, SD = 0.58 for mothers). The parents' perceived HPV severity (M = 4.11, SD = 0.82 for fathers; M = 4.06, SD = 0.92 for mothers) was slightly higher than HPV susceptibility (M = 3.95, SD = 0.84 for fathers; M = 3.74, SD = 0.93 for mothers). The perceived level of risks from HPV vaccines was low (M = 2.59, SD = 0.84 for fathers; M = 2.41, SD = 0.70 for mothers), while the perceived level of vaccine response efficacy was high (M = 4.08, SD = 0.80 for fathers; M = 4.01, SD = 0.71 for mothers). In terms of intention to vaccinate daughters, both fathers and mothers had high intention (M = 3.94, SD = 0.92 for fathers; M = 4.10, SD = 0.83 for mothers). The main resources of HPV vaccination information reported by the respondents are generally official media (M = 3.02, SD = 0.05) and We-media (M = 3.00, SD = 0.05).

**Table 1:** Descriptive statistics and independent samples *t*-test.

		**Mean**	**SD**	***F*-value**	***t*-value**	**df**	***p***
Information quality	F*	3.313	0.718	0.000	−1.184	352	0.237
	M	3.419	0.672				
Source credibility	F	4.621	0.455	5.159	1.986	136.2	*
	M	4.494	0.580				
HPV severity	F	4.105	0.824	0.660	0.348	352	0.728
	M	4.064	0.918				
HPV susceptibility	F	3.951	0.844	2.219	1.769	352	0.078
	M	3.738	0.929				
Secondary risk	F	2.591	0.838	2.008	1.915	352	0.056
	M	2.407	0.696				
Vaccine response efficacy	F	4.084	0.804	3.769	0.723	352	0.470
	M	4.014	0.707				
Intention to vaccinate	F	3.938	0.916	1.938	−1.398	352	0.163
	M	4.095	0.833				

F, Father; M, Mother; 1~5 indicated “strongly disagree,” “disagree,” “moderate,” “agree,” and “strongly agree”. *p < 0.05.

According to the results of the independent sample *t*-test, parents differed significantly only in the assessment of source credibility, as shown by the fact that fathers (M = 4.62, SD = 0.46) trusted online sources significantly more than mothers (M = 4.49, SD = 0.58). The rest of the variables did not show any difference between fathers and mothers.

### Examination of structural equation model

The model fit was assessed using the ratio of the chi-square value and the degree of freedom value, root mean square error of approximation (RMSEA), Tucker-Lewis index (TLI), and comparative fit index (CFI). The results showed a chi-square value of 2694.727, a degree of freedom of 819, and a ratio of 3.29. The TLI value of 0.838, CFI value of 0.848, and RMSEA value of 0.08 indicated an acceptable model fit.

The reliability of the measurement model was assessed using the composite reliability (CR), with a CR value above 0.7 indicating good intrinsic model quality (53). The results showed that the CR values were between 0.717 and 0.971. The construct validity of the latent variables was examined using convergent validity and discriminant validity. The convergent validity was evaluated by the average variance extracted value (AVE), which ranged from 0.560 to 0.880, higher than the recommended value of 0.5, indicating good convergent validity (Table 2).

**Table 2:** Composite reliability and convergent validity.

	**CR**	**AVE**
Information quality	0.971	0.739
Source credibility	0.836	0.720
HPV severity	0.925	0.711
HPV susceptibility	0.717	0.560
Secondary risk	0.931	0.630
Vaccine response efficacy	0.889	0.728
Intention to vaccinate	0.956	0.880

Discriminant validity was evaluated by comparing the square root of the AVE values with the absolute values of the correlation coefficients between different variables. The square roots of the AVE values in this study were all greater than the absolute values of the correlation coefficients between the corresponding latent variables and other variables (Table 3); the discriminant validity was therefore acceptable.

**Table 3:** Pearson correlation analysis and discriminant validity.

	**1**	**2**	**3**	**4**	**5**	**6**	**7**
1. Information quality	**0.860**						
2. Source credibility	0.162**	**0.849**					
3. HPV severity	0.211***	0.200**	**0.843**				
4. HPV susceptibility	0.249***	0.183**	0.077**	**0.748**			
5. Secondary risk	−0.066	−0.278***	−0.059**	−0.056*	**0.794**		
6. Vaccine response efficacy	0.139*	0.280***	0.073**	0.072**	−0.080**	**0.853**	
7. Intention to vaccinate	0.097**	0.203***	0.019	0.135*	−0.242***	0.513***	**0.938**

*p < 0.05, **p < 0.01, ***p < 0.001. The bold values indicate the square roots of the AVE values.

**Figure 2:**
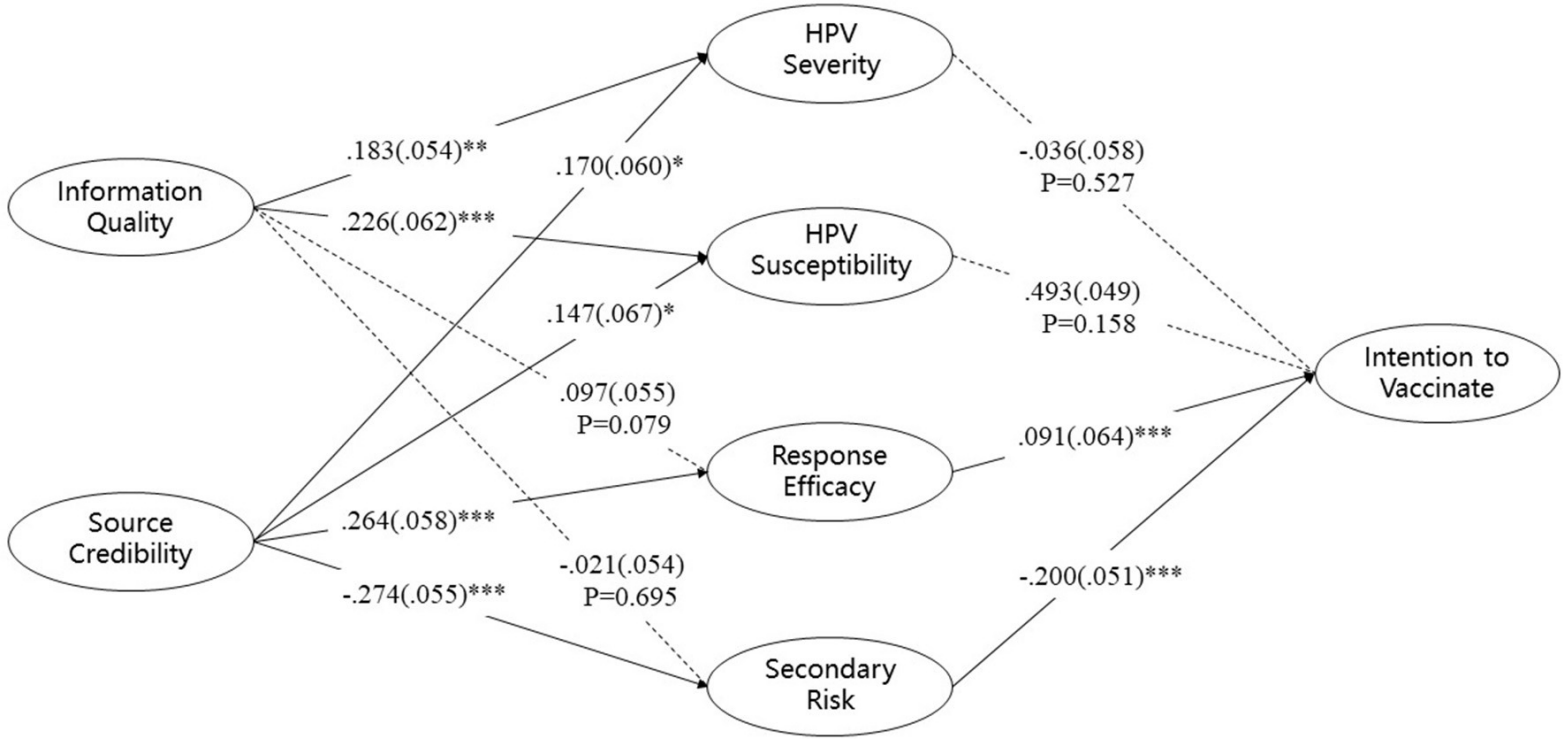
Standardized model coefficient diagram. *p < 0.05; **p < 0.01; ***p < 0.001.

The relationships between the different latent variables were assessed by examining the structural model. The hypothesized model and standardized model coefficients (standard errors) results are shown in Figure 2. Information quality was positively correlated with perceived HPV severity *(*β = *0.183, p*<*0.01)* and HPV susceptibility *(*β = *0.226, p*<*0.001)*, confirming hypotheses H2a and H2b. Source credibility was positively correlated with perceived HPV severity *(*β = *0.170, p*<*0.05)*, HPV susceptibility *(*β = *0.147, p*<*0.05)*, and vaccine response efficacy *(*β = *0.264, p*<*0.001)*, confirming hypotheses H3a, H3b, and H3c. Source credibility was negatively correlated with the perceived secondary risk of HPV vaccines *(*β = −*0.274, p*<*0.001)*, confirming hypothesis H3d. Perceived HPV vaccine response efficacy was positively correlated with parents' intention to vaccinate their children against HPV *(*β = *0.091, p*<*0.001)*, confirming hypothesis H1c. Perceived secondary risk was negatively correlated with parents' vaccination intention *(*β = −*0.200, p*<*0.001)*, confirming hypothesis H1d. The relationships between source credibility and vaccine response efficacy *(*β = *0.097, p* = *0.079)* and between source credibility and secondary risk *(*β = −*0.021, p* = *0.695)* were insignificant; hypotheses H2c and H2d were therefore not confirmed. The relationships between HPV severity *(*β = *- 0.036, p* = *0.527)*, HPV susceptibility *(*β = *0.493, p* = *0.158)*, and vaccination intention were not significant either, and therefore hypotheses H1a and H1b were not confirmed. Thus, 8 out of 12 paths in the hypothesized model were valid.

### Examination of mediating effects

Structural equation model testing was separately used to examine the mediating effects of vaccine response efficacy and secondary risk between source credibility and parents' intention to vaccinate their daughters; model 4 in PROCESS 3.2 was used, with a confidence interval of 0.05. If the lower and upper limits of the confidence interval do not contain 0, the mediating effect of the variables is confirmed.

As Table 4 shows, the confidence intervals on the vaccine response efficacy and secondary risk models do not contain 0. The indirect effects of source credibility on parents' intention to vaccinate their daughters through vaccine response efficacy and secondary risk were 0.163 and 0.097, respectively. Therefore, vaccine response efficacy and secondary risk had significant mediating effects in the path from source credibility to parents' intention to vaccinate.

**Table 4:** Mediating effect test.

**Model**		**Coefficient**	**Standard error**	**Lower limit (LLCI)**	**Upper limit (ULCI)**
Source credibility → Vaccine response	Direct effect	0.195	0.071	0.056	0.334
efficacy → Intention to vaccinate	Indirect effect	0.163	0.044	0.085	0.256
Source credibility → Secondary risk →	Direct effect	0.261	0.080	0.105	0.418
Intention to vaccinate	Indirect effect	0.097	0.031	0.045	0.164

## Discussion

This study examines how the information processing patterns of individuals affect their cognition and produce persuasive effects. The results show that the central route, represented by information quality, influences parents' assessment of HPV severity and susceptibility. The peripheral route, represented by source credibility, affects parents' perceptions of HPV severity, susceptibility, vaccine response efficacy, and secondary risk simultaneously. Perceptions of vaccine response efficacy and secondary risk are associated with parents' intention to have their daughters vaccinated against HPV. No significant relationships were found between HPV severity and susceptibility and vaccination intention. In addition, this paper shifts the persuasion effect of health information from media exposure to information processing. Previous studies have focused on the influence of media exposure on perceptions of vaccination and subsequent behaviors (54–56). This study focuses on how people's information processing routes affect their perceptions and behaviors after exposure to media, which is the core factor that affects the media effect.

### The central route affects parents' perceptions of HPV severity and susceptibility

When parents processed information about HPV and vaccines using the central route, information quality positively predicted their perceptions of HPV severity and susceptibility. This finding suggests that parents' thoughtful processing of vaccine-related information influences their assessment of HPV severity and susceptibility, and that higher information quality is associated with stronger perceptions of virus severity and susceptibility. This is consistent with previous research (57), which has shown that text-only warnings (central route) can influence lottery gamblers' perceived threat severity and susceptibility. Warnings about the threat of gambling also inspire more fear when gamblers perceive themselves as more susceptible to threats, compared to merely perceiving the severity of the negative effects of gambling.

However, information quality did not predict parents' perceptions of vaccine protection and risks, i.e., the central route failed to influence parents' perceptions of protective behaviors. This contradicts a previous study of the factors that influence the level of employee engagement in information security protective behaviors (58). That study found that the higher the information quality, the more likely employees are to develop good cognitive attitudes toward recommended information security behaviors. We think that Limited Capacity Model of Motivated Mediated Message Processing (LC4MP) can help understand the discrepancy (59). When people are dealing with knowledge beyond their comprehension, they may only remember it instead of understanding it. Because of the scientific nature of vaccine knowledge and the political and economic interests behind vaccination, information about HPV vaccines is often complex for parents to understand. Our interviews also confirmed this explanation:

First, vaccine knowledge is more complex and multidimensional than perceptions of virus severity and susceptibility. High-quality information may not give “laypeople” a comprehensive knowledge of vaccines, and it is difficult to obtain systematic explanations of vaccines from the news. Respondents said, “There are too many medical terms in the news about vaccines, which are quite obscure”; “I don't know much about vaccines, mRNA, or protein.”

Second, most parents were born in the 1970s and remembered the vaccine incidents widely reported by the media. It is difficult to dispose of stereotypes even with high-quality news explanations. “When I was a child, there were reports that the hepatitis A vaccines seemed to have killed a few elementary school students. Vaccines involve injecting the virus into bodies after all, so I still have to be careful.”

Third, vaccines cannot be disentangled from other areas of society, such as the political and commercial spheres. Vaccine information is a complex mediator in the knowledge production of biosciences, market organizations, and other social groups (60). Social agents with different interests spread information with different focuses based on their perspectives, making it difficult for parents who lack professional knowledge to obtain clear vaccine perceptions from the media, even if the information is of high quality.

### The peripheral route influences parents' perceived HPV severity, HPV susceptibility, vaccine response efficacy, and secondary risks

The level of trust in the information source can simultaneously influence parents' perceptions of HPV severity, susceptibility, vaccine response efficacy, and secondary risk, suggesting that the peripheral route significantly influences parents' perceptions of the virus and vaccines. This result is similar to the findings of previous research (61) into the effect of fear appeals on information security compliance behavior, which found that stronger perceptions of threat severity, susceptibility, and response efficacy will develop when the rhetoric about fear appeals (peripheral route) aligns with the audience's rhetorical preferences.

Further, when the media that parents trust release health information, their attitudes toward viruses and vaccines may be influenced even though they have not processed the information in depth. “*People's Daily* says it works, so it should work, right?” “Dr. Wenhong Zhang also took his daughter to get HPV vaccines, so it should be safe.” Therefore, compared to the central route, rich peripheral cues help parents form correct perceptions of HPV and vaccines.

### Perceptions of vaccines, not perceptions of HPV, affect intention to vaccinate

Overall, this study suggests that perceptions of HPV are not significantly associated with parents' intention to vaccinate their daughters. Instead, perceptions of the vaccines predict parents' intention. Specifically, although parents recognize HPV as dangerous and easily transmitted, it does not mean that they will have their daughters vaccinated. Parents are only likely to immunize their daughters when they perceive HPV vaccines to be an effective, low-risk defense against the virus. In Chinese folk tradition, HPV is associated with female infidelity, and HPV infection has been stigmatized as a “sign of dishonor.” Traditional gender attitudes are deeply rooted, and it would be a family scandal if a daughter were infected with HPV.

The interviews reveal that while parents believed HPV is a serious, easily transmitted disease, they also believe the chances of their own daughters being infected to be extremely low. Respondents said, “My daughter is clean, so it is impossible for her to get HPV”; “HPV is sexually transmitted, but my daughter is very well behaved.” PMT provides a multiplicative model for the Chinese context. If any of the four components is missing, the protection motivation will be weakened and the intention to vaccinate will be reduced. One of the interviewed parents said, “My daughter is unlikely to be infected with HPV. The vaccine is a virus after all, it is safer not to vaccinate. Human genes can be changed by the vaccine”; another remarked, “The HPV vaccines are given to those who have frequent sex.” This shows that parents will only increase their intention to vaccinate their daughters if they believe that their daughters are at risk for HPV infection and that the vaccine is effective and safe.

### Mediating effects of HPV vaccine response efficacy and secondary risks

This study shows that perceived vaccine response efficacy and perceived secondary risks have significant mediating effects on the relationship between source credibility perceptions and vaccination intention. This suggests that after processing vaccine information through the peripheral route, parents consider having their daughters vaccinated when they believe the vaccines are effective and safe. The mediating effects demonstrate the existence of a “media-cognition/motivation-behavioral intention” influence model. Parents immersed in the information sources reconsider and change their perceptions of the vaccines after processing the information through central and peripheral routes, and may then adopt vaccination behavior.

## Limitations

This study has some shortcomings. First, we used the self-reporting method to obtain the subjects' behavioral data, which is subjective (62). The scales of some variables used mature foreign scales that lacked localization. If future studies use more scales validated by local data, this will improve the measures' accuracy. Second, this study examined the mediating effects using cross-sectional data. However, cross-sectional surveys cannot determine causality, reducing the reliability of the mediating effects when the causal chain is unknown. If future studies use data tracking or multi-point measurement methods, the mediating effect can be examined more rigorously. Thirdly, this study only used information quality to represent the central route and source credibility to represent the peripheral route. Future studies can use other relevant variables to increase the validity of the examination of the ELM. Finally, we did not introduce the response cost from PMT. However, in the Chinese context, psychological costs related to emotions and social norms may also be important in parents' vaccination decision-making for their daughters. We look forward to future studies that measure the response costs of vaccination from a psychological perspective.

## Conclusion

This study shows that the central and peripheral routes affect different aspects of protection motivation. The central route affects parents' perceptions of HPV severity and susceptibility and the peripheral route influences parents' perceptions of HPV severity, HPV susceptibility, vaccine response efficacy, and secondary risks. Therefore, in addition to improving the quality of health information, the peripheral route should also be used to change parents' perceptions. Studies have shown that pictures and photos, price, and narrative approaches are also considered rich peripheral cues (10). As part of vaccine promotion, aside from the extensive release of health information by authoritative media, the release of vaccination photos, public immunization evaluations, and case narratives can correct parents' perceptions of HPV and vaccines. These peripheral cues can make up for the deficiency of the central route in influencing vaccine awareness. HPV vaccine information currently does not effectively use peripheral cues, and news on social media platforms is highly homogeneous, with single communication methods, little use of pictures and cartoons, more jargon, and less personalized narratives. Such mechanical, uninvolving science- and fact-based information does not attract audiences or generate sustained social attention (63).

In addition, perceptions of HPV do not affect parents' intention to vaccinate their daughters. Even if Chinese parents fully learn about the severity and infectiousness of HPV, they still believe that their daughters will not be infected due their conservative attitudes. Nonetheless, the age of young women's first sexual experience in China is now 15 years old, and sexual attitudes tend to be open and tolerant generally (64). Reducing the traditional stigmatization of female sexuality and improving parents' understanding of the new generation's sexual attitudes will increase parents' intention to have their daughters vaccinated against HPV.

## Data availability statement

The datasets generated and analysed during the current study are not publicly available due to the consideration of participants' privacy, but are available from the corresponding author on reasonable request.

## Ethics statement

The studies involving human participants were reviewed and approved by the Research Ethics Committee of Zhongnan University of Economics and Law. The patients/participants provided their written informed consent to participate in this study.

## Author contributions

Research methodology: QW, FZ, and WZ. Formal analysis: FZ and QW. Survey and data collation: CT and WZ. Writing–original draft preparation: FZ. Writing–review and editing: QW. Supervision: WZ. Funding acquisition: QW and WZ. All authors have reviewed and agreed to the published version of the manuscript.

## Funding

This study was supported by the Ministry of Education of China, Humanities and Social Science Foundation Research Fund (No. 21YJC760081).

## Conflict of interest

The authors declare that the research was conducted in the absence of any commercial or financial relationships that could be construed as a potential conflict of interest.

## Publisher's note

All claims expressed in this article are solely those of the authors and do not necessarily represent those of their affiliated organizations, or those of the publisher, the editors and the reviewers. Any product that may be evaluated in this article, or claim that may be made by its manufacturer, is not guaranteed or endorsed by the publisher.
